# JNK signalling mediates aspects of maternal immune activation: importance of maternal genotype in relation to schizophrenia risk

**DOI:** 10.1186/s12974-019-1408-5

**Published:** 2019-01-28

**Authors:** Rebecca L. Openshaw, Jaedeok Kwon, Alison McColl, Josef M. Penninger, Jonathan Cavanagh, Judith A. Pratt, Brian J. Morris

**Affiliations:** 1 Institute of Neuroscience and Psychology, West Medical Building, College of Medical, Veterinary and Life Sciences, University of Glasgow, Glasgow, G12 8QQ UK; 2 Strathclyde Institute of Pharmacy and Biomedical Sciences, University of Strathclyde, Glasgow, UK; 3 Institute of Inflammation and Immunity, University of Glasgow, Glasgow, UK; 4 IMBA, Institute for Molecular Biotechnology of the Austrian Academy of Sciences, Vienna, Austria

**Keywords:** Neurodevelopment, Chemokine, ip-10, RANTES, sdf-1, TLR3, Poly(IC), Poly(I:C)

## Abstract

**Background:**

Important insight into the mechanisms through which gene-environmental interactions cause schizophrenia can be achieved through preclinical studies combining prenatal immune stimuli with disease-related genetic risk modifications. Accumulating evidence associates JNK signalling molecules, including MKK7/*MAP2K7*, with genetic risk. We tested the hypothesis that *Map2k7* gene haploinsufficiency in mice would alter the prenatal immune response to the viral mimetic polyriboinosinic-polyribocytidylic acid (polyI:C), specifically investigating the impact of maternal versus foetal genetic variants.

**Methods:**

PolyI:C was administered to dams (E12.5), and cytokine/chemokine levels were measured 6 h later, in maternal plasma, placenta and embryonic brain.

**Results:**

PolyI:C dramatically elevated maternal plasma levels of most cytokines/chemokines. Induction of IL-1β, IL-2, IL-10, IL-12, TNF-α and CXCL3 was enhanced, while CCL5 was suppressed, in *Map2k7* hemizygous (Hz) dams relative to controls. Maternal polyI:C administration also increased embryonic brain chemokines, influenced by both maternal and embryonic genotype: CCL5 and CXCL10 levels were higher in embryonic brains from *Map2k7* dams versus control dams; for CCL5, this was more pronounced in *Map2k7* Hz embryos. Placental CXCL10 and CXCL12 levels were also elevated by polyI:C, the former enhanced and the latter suppressed, in placentae from maternal *Map2k7* Hzs relative to control dams receiving polyI:C.

**Conclusions:**

The results demonstrate JNK signalling as a mediator of MIA effects on the foetus. Since both elevated CXCL10 and supressed CXCL12 compromise developing GABAergic interneurons, the results support maternal immune challenge contributing to schizophrenia-associated neurodevelopmental abnormalities. The influence of *Map2k7* on cytokine/chemokine induction converges the genetic and environmental aspects of schizophrenia, and the overt influence of maternal genotype offers an intriguing new insight into modulation of embryonic neurodevelopment by genetic risk.

**Electronic supplementary material:**

The online version of this article (10.1186/s12974-019-1408-5) contains supplementary material, which is available to authorized users.

## Background

Schizophrenia, which affects around 1% of the population, is caused by the combined action of genetic and environmental risk factors. In recent years, there has been considerable progress in understanding the genetic contribution to disease risk. For most patients, this derives from the interaction of a large number of common genetic variants, all individually contributing a small increase in risk [1]. In other patients, rare variants have a proportionately larger effect. Many of the variants associated with schizophrenia, both common and rare, can be functionally aligned with glutamatergic signalling mechanisms [2–5]. The proteins involved encompass structural molecules of the post-synaptic specialisation (such as Arc and PSD95) and downstream signalling molecules.

There is a substantial body of research indicating that environmental risk factors that act during the pre-, peri- and early postnatal period are also important in the pathogenesis of schizophrenia in adult offspring [6, 7]. Stressful events, such as psychological trauma, maternal malnutrition, gestational infection and other obstetric complications, cause physiological changes in the developing foetal environment, disturbing the normal course of brain development and inducing structural and functional brain abnormalities which emerge later, in adult life [6–8]. In particular, maternal infection and the associated inflammatory response in the mother (and possibly in the developing foetal brain) have been a significant focus of investigation. The effect of maternal immune activation (MIA) on offspring in preclinical rodent models (reviewed in [9]) continues to provide insight into the potential pathogenic mechanisms involved in prenatal infection and the neurodevelopmental hypothesis of schizophrenia [10].

MIA using polyriboinosinic-polyribocytidylic acid (polyI:C), a double-stranded RNA virus mimetic, is a particularly well-studied method of immune activation [11–13]. PolyI:C is recognised by toll-like receptor 3 (TLR3) [14], and a strong inflammatory response is initiated that brings cells to the site of infection in order to help kill the invading pathogen. This occurs via the activation of kinase signalling cascades, most prominently the JNK and NF-κB pathways [15, 16], subsequently upregulating genes coding for pro- and anti-inflammatory mediators such as cytokines, chemokines and colony-stimulating factors (CSFs) [17, 18].

PolyI:C administration to gestating rodents has repeatedly been shown to induce molecular, structural, physiological and behavioural changes related to schizophrenia in adult offspring [12, 13]. Reported molecular changes in the offspring include altered microglial staining, altered dopamine metabolism in the striatum, alterations in glutamic acid decarboxylase-67 (GAD-67) expression and reduced parvalbumin expression in the prefrontal cortex [19–21].

These findings have provided a new impetus to attempts to identify rodent models of aspects of schizophrenia neurobiology with improved construct validity—the combination of genetic risk factors with developmental immune challenge. Of the few studies so far reported, mutations in the DISC1 gene have been combined with prenatal [22] or early postnatal [23] administration of polyI:C. Another group [24] combined mice with a hemizygous functional deletion of the neuregulin (*Nrg1*) gene with maternal polyI:C exposure. However, the genetic variants studied in this respect to date have no obvious relationship with the environmental stimulus. The recent evidence implicating the JNK signalling pathway, and specifically the kinases involved in JNK activation, such as MKK7 (*MAP2K7)* [25], ULK4 [26], and VRK2 and TAOK2 [4, 27], in genetic risk for schizophrenia [2, 28] is of particular interest, since MKK7-JNK signalling is not only involved in glutamatergic signalling in the CNS [29], but is also believed to mediate aspects of the innate immune response [30]. The potential link between genetic risk and environmental risk is clearly intriguing.

Mice hemizygous for a functional deletion of the *Map2k7* gene (*Map2k7* Hz mice) show reduced CNS expression of MKK7, along with subtle cognitive deficits characteristic of patients with schizophrenia, including an inability to sustain attention in cognitive tasks [31]. This study tests the hypothesis that *Map2k7* Hz mice also show an altered cytokine/chemokine response to maternal immune challenge. Gestating dams at E12.5 are exposed to polyI:C and the immune response profile determined in maternal plasma and brains of the developing embryos.

## Methods

### Maternal immune activation

Mice hemizygous for a functional deletion of the *Map2k7* gene (*Map2k7* Hz mice) [32], and wild-type (WT) C57Bl6 mice (littermates with *Map2k7* Hz mice), were used in the experiment. Mice were time mated according to the combinations outlined in Table 1. Mouse pairs were put together at 5 p.m. and separated in the morning the next day. If they had conceived, this was taken as embryonic day 0.5. Female mice were weighed and monitored for 12 days.

**Table 1:** Mating combinations. Four pairs in each group were successfully time mated, totalling 16 pairs. Each pair produces WT and *Map2k7* Hz embryos

Breeding	Treatment	Maternal plasma samples	Litters	Independent WT embryonic brain samples	Independent Hz embryonic brain samples
Female WT × Male *Map2k7* Hz	Saline (vehicle)	4 × WT	4	4	4
Female WT × Male *Map2k7* Hz	PolyI:C (20 mg/kg sc.)	4 × WT	4	4	4
Female *Map2k7* Hz × Male WT	Saline (vehicle)	4 × Hz	4	4	4
Female *Map2k7* Hz × Male WT	PolyI:C (20 mg/kg sc.)	4 × Hz	4	4	4

Female mice weighed 22.1 ± 0.34 g on average at the start of the experiment and 28.7 ± 0.77 g on average when 12.5 days pregnant. All mice were aged 12.23 ± 0.47 weeks at the point of conception. Mice were singularly housed (when not paired) in a temperature and humidity controlled room with a 12-h light/dark cycle (lights on at 07:00) in accordance with the Animals (Scientific Procedures) Act 1986. Pregnant dams were weighed and given either 20 mg/kg at 2 ml/kg polyI:C or 2 ml/kg saline on embryonic day 12.5. This dose has previously been shown to induce long-lasting behavioural and pharmacological changes in mouse offspring [33] and is arguably the optimal dose that causes MIA [34]. With respect to brain development [35] and brain gene expression [36], it has been argued that embryonic day 12.5 may be equivalent to approximately the 54th day (7.8th week) of gestation for humans. Therefore, embryonic day 12.5 is the murine equivalent of three quarters of the way through trimester 1 in humans, a period where the developing nervous system is particularly vulnerable to maternal infection and most associated with increased incidence of schizophrenia [37]. All injections were given subcutaneously to avoid accidental injection into an embryo.

### Protein extraction for ELISA and Luminex

Six hours following the polyI:C or saline injection, the pregnant dam was injected with a lethal dose (0.1 ml) of pentobarbital sodium (Euthatal, Merial Animal Health Ltd.) and trunk blood was collected via cardiac puncture into an EDTA-coated syringe. The blood was injected into an EDTA-coated 1.5-ml Eppendorf tube containing an additional 80 μl EDTA and shaken. Following centrifugation at 10,000*g* at 4 °C for 10 min, the supernatant (plasma) was frozen at − 80 °C until ELISA or Luminex assay were conducted.

The placentae and embryos were carefully removed, and the brain, placentae and a small amount of tissue (for genotyping) kept at − 80 °C until required. Within each litter, embryonic brain samples were pooled for genotype after genotyping, so that each litter yielded one WT and one *Map2k7* Hz pooled brain extract. The brain tissue was homogenised manually in 275 μl lysis buffer (1x PBS with 0.1% Triton X-100 (Sigma), 5 μM EDTA (GIBCO) and proteinase inhibitors (Sigma)) and then centrifuged at 8000*g* for 10 min at 4 °C. The supernatant containing the protein was then halved (~ 110 μl each): one for ELISA and one for Luminex. Embryonic brain protein concentrations were determined by bicinchoninic acid (BCA) assay (Merck) according to the manufacturer’s instructions.

### Luminex assay

The concentration of 20 cytokines, chemokines and colony-stimulating factors were determined simultaneously in maternal plasma and embryonic brain tissue supernatant using a mouse cytokine magnetic 20-plex assay according to the manufacturer’s instructions (Invitrogen: LMC0006M). Samples were analysed in duplicate: the coefficient of variation (% CV) was checked; a plate was considered acceptable if the mean CV < 15% and if not more than 20% of duplicates have CV > 25%. The mean CV for each plate was 5.25% and 6.74%, and the percentage of duplicates which have a CV > 25% was 1.35% and 3.12% for each plate, respectively, which was well within this range. Further details are provided in Additional file 1. Embryonic brain concentrations were normalised to total protein via the BCA assay.

### ELISA

Since CCL5 is a relatively well-characterised component of the innate immune response, but is not represented on the Luminex assay employed for the other cytokines/chemokines, CCL5 levels in maternal plasma and embryonic brain tissue supernatant were measured by enzyme-linked immunosorbent assay (ELISA). Additional measurements of CXCL10 and CXCL12 levels in placenta were also performed by ELISA. Mouse Quantikine® ELISAs, MMR00, MCX100 and MCX120 (R&D Systems, Abingdon, UK), were utilised according to the manufacturer’s instructions. Fifty microlitre of assay diluent (provided) and 50 μl of the diluted standards and samples (undiluted) were assayed in duplicate. The optical density was then read by a plate reader (Multiskan Spectrum, Thermo Fisher). Embryonic brain and placenta concentrations were normalised to total protein as established by the BCA assay.

### Low level processing and statistical analysis

For the maternal plasma measurements, 14 of the 21 immune response/growth factor molecules analysed were within a detectable range and met criteria for inclusion in analyses (see Additional file 1); for the embryonic brain measurements, five were detectable. This is most likely because the levels of immune molecules are substantially lower in embryonic brain tissue than in maternal blood plasma [34] and/or not present in embryonic brain at detectable levels at this stage of development [17].

All statistical analyses were carried out using Minitab 17 Statistical Software. For maternal plasma, each cytokine was analysed separately by a two-way ANOVA with maternal genotype (WT or *Map2k7* Hz) and drug (saline or polyI:C) as between-subjects factors. For embryonic brain, each cytokine was analysed separately by a three-way ANOVA with maternal genotype (WT or *Map2k7* Hz), embryonic genotype (WT or *Map2k7* Hz) and drug (saline or polyI:C) as between-subjects factor and each litter nested within maternal genotype and drug. Two-way pairwise comparisons were made between factors using Tukey’s method. Data are presented as mean ± standard error of the mean (SEM), and results were considered significant if *p* < 0.05. Some of the cytokine levels measured returned zero values, because of levels below detection only in saline groups. Therefore, some data groups did not show equal variances and normal distribution. However, data within polyI:C treatment groups alone showed normal distributions. Where significant departure from normality was detected, a box-cox transformation of the data was employed. Where substantial departure from Gaussian distribution was noted, ANOVAs were performed only on the polyI:C groups, or by non-parametric analysis, for additional confirmation.

## Results

**Fig. 1:**
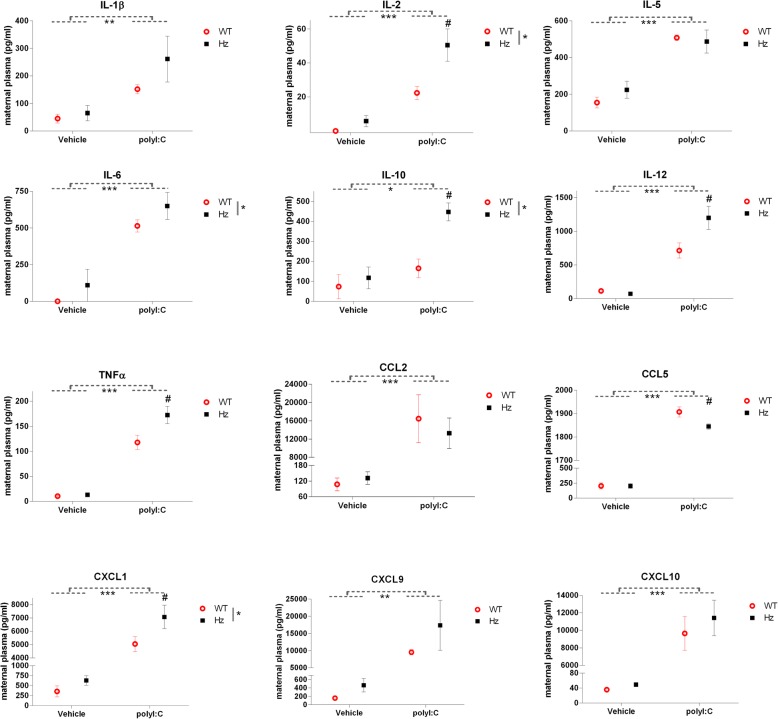
Cytokine levels in maternal plasma as measured by Luminex multiplex panel or ELISA (CCL5 only). All detectable cytokines/chemokines were elevated in the plasma of mothers that received polyI:C compared to those that received saline. Plasma IL-2, IL-10, IL-12, TNF-α and CXCL1 were significantly more elevated in *Map2k7* Hz mice than they were in WT mice, following polyI:C administration compared to saline. Results shown are mean ± SEM, *N* = 4/group. Data were analysed by two-way ANOVA with maternal genotype (WT or *Map2k7* Hz) and treatment (saline or polyI:C) as factors. *F* values are provided in Additional file 1. *p < 0.05, **p < 0.01, ***p < 0.001 (main effect—ANOVA). ^#^*p* < 0.05 vs WT, the same treatment (post-hoc Fisher’s test)

Maternal plasma from mice injected with polyI:C had increased levels of most immune molecules detected compared to those injected with saline. Of the 12 cytokines/chemokines tested that were detectable in maternal plasma, all were elevated following polyI:C administration compared to saline: IL-1β, IL-2, IL-5, IL-6, IL-10, IL-12, TNF-α, CCL2, CCL5, CXCL1, CXCL9 and CXCL10 (Fig. 1). Interestingly, the levels of IL-2, IL-6, IL-10, TNF-α and CXCL1 were significantly higher in plasma from *Map2k7* Hz mice compared to WT controls overall, independent of treatment. Furthermore, the levels of IL-2, IL-10, IL-12, TNF-α and CXCL1 were significantly higher in plasma from *Map2k7* Hz mice compared to WT controls after polyI:C treatment. Conversely, the levels of CCL5 were significantly lower in plasma from *Map2k7* Hz mice after polyI:C treatment (Fig. 1).

### CCL5 and CXCL10 were elevated in embryonic brain following polyI:C

**Fig. 2:**
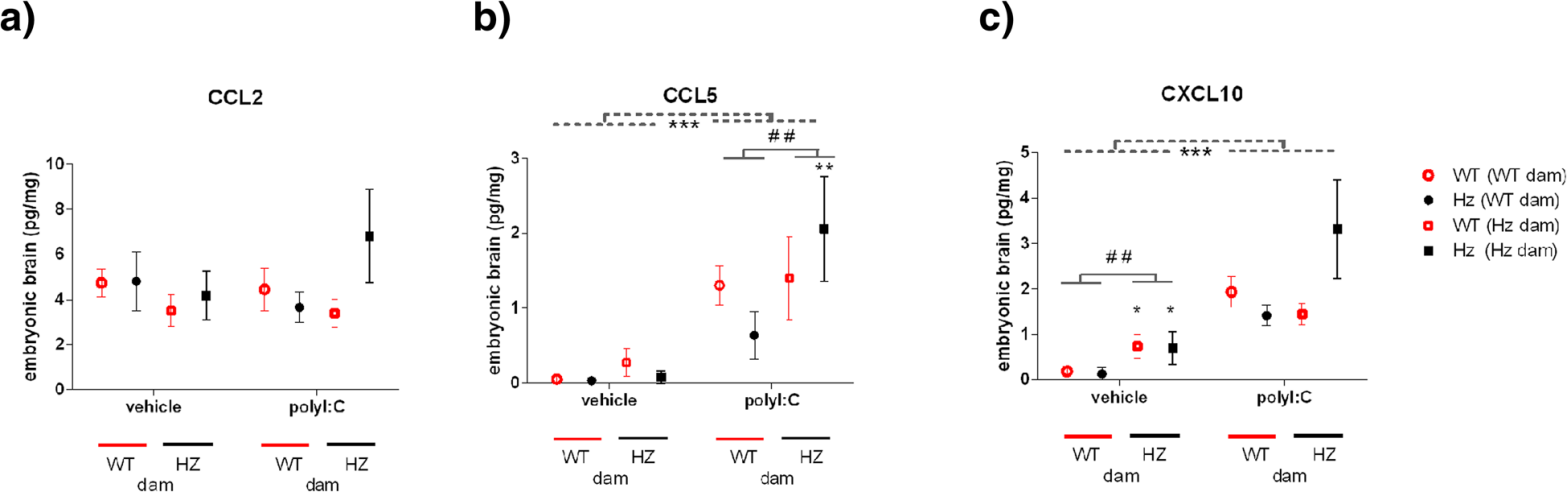
Chemokine levels in embryonic brain as measured by Luminex Multiplex panel or ELISA (CCL5 only). CCL2 levels were unaffected by genotype or treatment (**a**). Overall, CCL5 (**b**) and CXCL10 (**c**) levels were significantly increased in the brains of embryos whose mother had been exposed to polyI:C, compared to brain levels in embryos whose mother had received saline. Results shown are mean ± SEM. Data analysed by three-way ANOVA with embryonic genotype, maternal genotype, and treatment as factors. *F* values are provided in Additional file 1. *N* = 4/group. ***p < 0.001 effect of treatment (ANOVA); ^##^*p* < 0.01 as shown; *p < 0.05, **p < 0.01 vs WT dam, the same treatment and embryo genotype (post-hoc Fisher’s test)

None of the cytokines tested were found to be above the threshold for detection in embryonic brain. Of the chemokines analysed, three were within a detectable range and met criteria for inclusion in analyses: CCL2, CCL5 and CXCL10. Interestingly, under basal conditions, the levels of CXCL10 in embryonic brain were higher with *Map2k7* Hz mothers than with WT mothers (*p* < 0.01) (Fig. 2c). Equally, embryos from *Map2k7* Hz dams had increased CCL5 brain levels overall compared to embryos from WT dams (*p* = 0.031) (Fig. 2b), illustrating the operation of maternal genotype effects on foetal brain chemokine expression.

Analysis suggested that, overall, CCL2 was not elevated in embryonic brain in response to maternal polyI:C exposure (Fig. 2a). However, CCL5 and CXCL10 levels were significantly increased in the brains of embryos whose mother had been exposed to polyI:C compared to the brains of embryos whose mother had been given saline (Fig. 2b, c). In addition, after polyI:C administration, *Map2k7* Hz embryos from *Map2k7* Hz mothers had a greater induction of brain CCL5 levels compared to *Map2k7* Hz embryos from WT mothers (Fig. 2b).

**Fig. 3:**
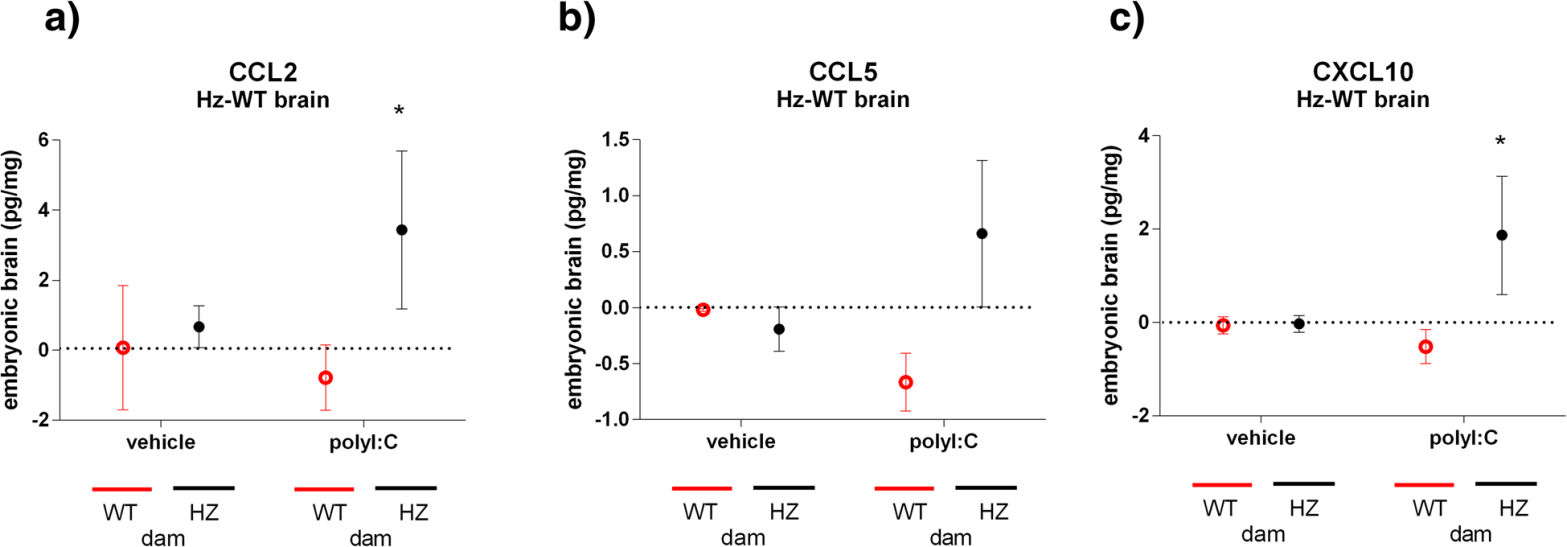
Within-litter differences for chemokine levels in embryonic brain as measured by Luminex Multiplex panel or ELISA (CCL5 only). Overall, CCL2 (**a**) and CXCL10 (**c**) brain differences between embryo genotypes were significantly altered after polyI:C in Hz dams—*p = 0.04 (CCL2) or *p* = 0.03 (CXCL10)—vs WT dams, same treatment (Mann-Whitney test). Results shown are mean ± SEM, *N* = 4/group

When within-litter differences due to foetal genotype were compared directly, by subtracting the level in the pooled WT foetal brains from the level in the pooled *Map2k7* Hz foetal brains, for each litter, the data suggested that, following maternal polyI:C exposure, there was an interaction between maternal and foetal genotype, resulting in a relatively greater induction of foetal brain CCL2 and CXCL10, in *Map2k7* Hz offspring from *Map2k7* Hz dams (Fig. 3).

**Fig. 4:**
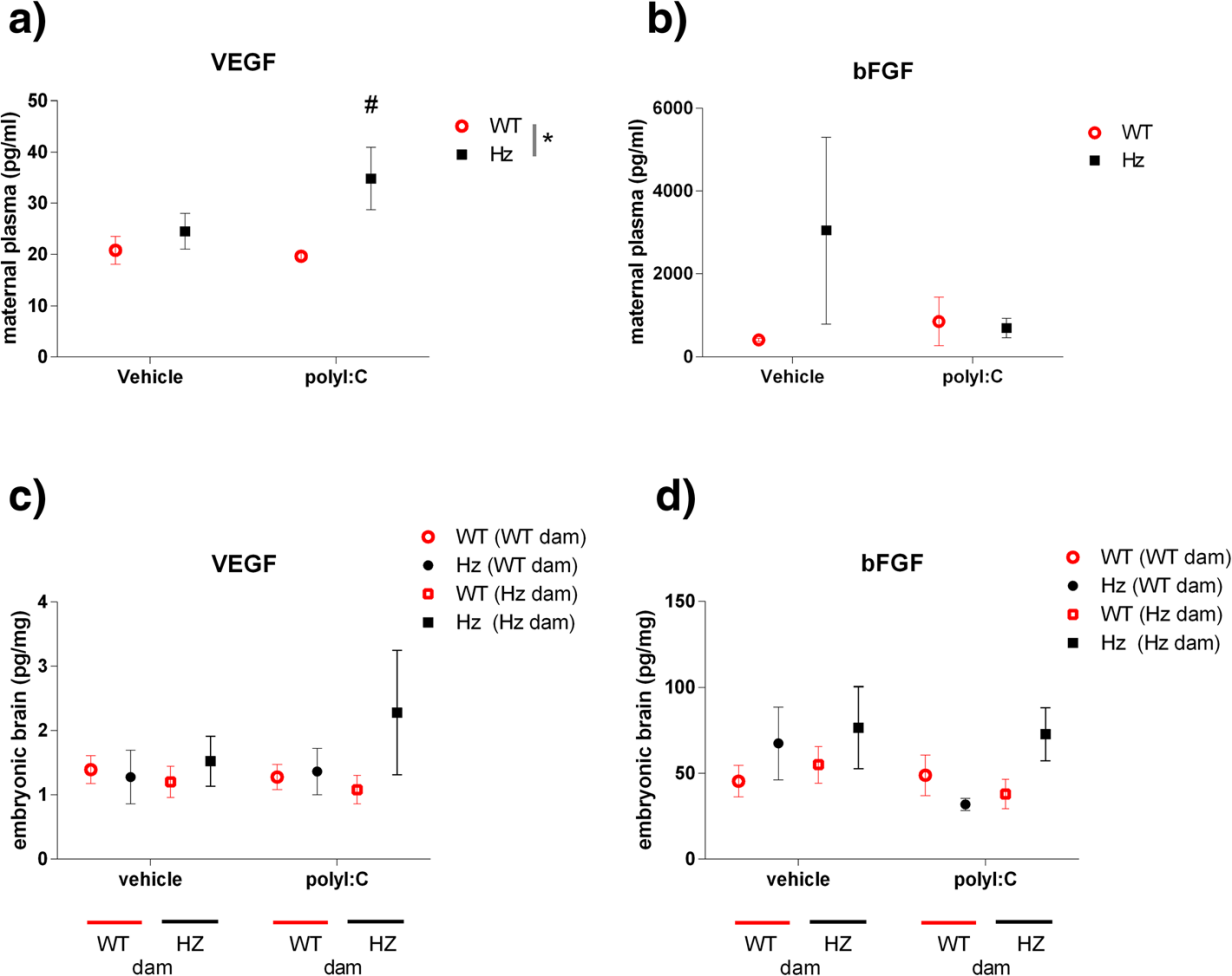
Growth factor levels in **a**, **b** maternal plasma, and **c**, **d** embryonic brain. VEGF levels (**a**, **c**) and basic FGF levels (**b**, **d**) were not significantly affected, in either maternal plasma or brain, by polyI:C administration. Results shown are mean ± SEM, *p < 0.05 (ANOVA); ^#^*p* < 0.05 vs WT dams, same treatment (post-hoc Fisher’s test); *F* values are provided in the Additional file 1. *N* = 4/group

The growth factor basic FGF was not elevated following polyI:C administration, either in maternal plasma (Fig. 4b) or in embryonic brain (Fig. 4d). VEGF levels were raised in *Map2k7* Hz dam plasma relative to WT controls (Fig. 4a), but were not significantly affected in embryonic brain (Fig. 4c).

As the main barrier to immune communication between factors in the maternal blood and in the foetus, the placenta is potentially important in the effect of maternal immune activation on the foetal brain. Indeed, preeclampsia is one of the clearest risk factors for future development of schizophrenia in offspring [37, 38]. We therefore additionally monitored the expression of two key mediators in the placenta from these mice: CXCL10 (originally IP-10) and CXCL12 (originally SDF-1) are both implicated in preeclampsia and in the innate immune response to *Toxoplasma gondii* [39–43]. In addition, increased CXCL10 [44] and decreased CXCL12 have been linked to impaired migration and function of cortical GABAergic interneurons [45, 46] and so may have particular relevance to schizophrenia risk.

**Fig. 5:**
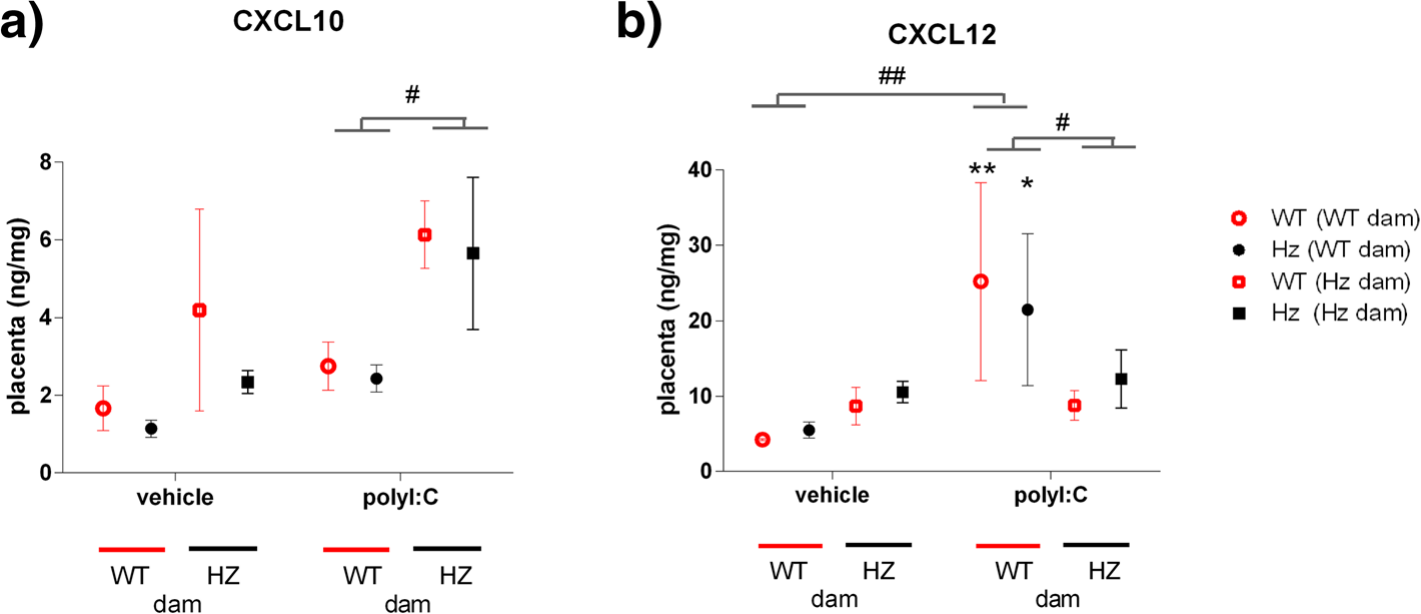
CXCL10 and CXCL12 levels in placenta. CXCL10 levels in placental tissue, measured by ELISA, were increased by polyI:C administration in placentae from *Map2k7* Hz dams but not WT dams, irrespective of the embryonic genotype (**a**). Conversely, CXCL12 levels were increased by polyI:C administration in placentae from WT dams but not *Map2k7* Hz dams in placental tissue, again irrespective of the embryonic genotype (**b**). Results shown are mean ± SEM, *N* = 4–7/group. ^#^*p* < 0.05, ^##^*p* < 0.01 as shown, *p < 0.05, **p < 0.01 vs vehicle-treated group, same dam and embryo genotype (post-hoc Fisher’s test); *F* values are provided in the Additional file 1

We found that there was a clear interaction of the effect of polyI:C with maternal genotype, such that, after the immune challenge, placental CXCL10 was elevated to a greater extent in *Map2k7* Hz dam placentae. In contrast, CXCL12 levels were elevated by polyI:C administration only in placentae from WT, but not in *Map2k7* HZ dams. There was no significant influence of the embryonic genotype (Fig. 5). Interestingly, there was also no detectable correlation between placental and embryonic brain levels of CXCL10 (in Additional file 1: Figure S1).

## Discussion

The results obtained in this study reveal that the immune response of *Map2k7* Hz mice differs from that of control mice, following maternal exposure to the viral mimetic polyI:C. This implicates JNK signalling in aspects of MIA. We confirm previous reports that MIA using polyI:C triggers an immune response in the foetal brain. We also present evidence that the levels of immune mediators in the foetal brain are affected by the maternal genotype as well as the embryonic genotype.

### Maternal plasma cytokine/chemokine response

MIA with polyI:C in gestating rodents is a popular experimental paradigm [11, 47]. However, the profile of the immune response to polyI:C in the maternal plasma has seldom been documented. Meyer et al. [20] reported increased levels of IL-1β, IL-6, IL-10 and TNF-α in maternal plasma 6 h after polyI:C administration in mice at E9 or E16. One other study assessed a relatively full profile of the immune response and reported that the same dose of polyI:C (20 mg/kg) to that used here increased levels of IL-6, IL-10, IL-12, IL-13, IL-15, TNF-α, IFN-γ, CCL2, CCL3, CCL5, CXCL1, CXCL9, CXCL10, VEGF and GM-CSF in maternal serum 6 h following exposure at E16 [17]. Our findings in maternal plasma correspond to those of Arrode-Brusés and Brusés [17] for the majority of immune molecules (increased levels of IL-6, IL-10, IL-12 and TNF-α, CCL2, CCL5, CXCL1, CXCL9, CXCL10). We also found an increase in IL-1β, IL-5 and IL-2, which they did not assay, and we did not observe their reported increase in VEGF. Overall, however, our findings are closely matched and show that the levels of a broad spectrum of immune molecules are increased following administration of a viral mimetic. The minor differences may be a result of the differences in foetal age [20], as there are fluctuations in the functioning of the maternal host’s immune system as pregnancy progresses [48]. It is also worth noting that many of the cytokines altered in the maternal serum are also elevated in the blood of patients with schizophrenia, including IL-1β, IL-2, IL-6, IL-12 and TNF-α [49–53].

### Differential upregulation of maternal plasma cytokines/chemokines in *Map2k7* Hz and WT mice

Twelve cytokines/chemokines were increased in maternal plasma following polyI:C exposure. Of these, IL-2, IL-10, IL-12, TNF-α and CXCL1 were all increased to a significantly greater extent in maternal plasma of *Map2k7* Hz mice compared to WT mice. In contrast, the elevation of CCL5 was suppressed in *Map2k7* Hz mice. Our results imply that maternal MKK7-JNK signalling acts to promote CCL5 induction and dampen the activation of IL-2, IL-10, IL-12, TNF-α and CXCL1.

Traditionally, JNK signalling is linked to positive transcriptional effects on immune response genes [54]. The elevated response for many factors due to haploinsufficiency for *Map2k7* is therefore at first sight surprising. However, JNK activation suppresses IL-2, IL-4 and IL-10 production in T cells [55], an effect mediated by MKK7. Hence, an enhanced response for IL-2 and IL-10 to polyI:C could be predicted. It may be that positive transcriptional responses to immune challenge are mediated predominantly via MKK4 (which can activate both JNKs and p38s) rather than MKK7 (which is specific for MKK7) [56]. Since MKK7 and MKK4 are seldom studied selectively, it could be very intriguing to follow-up this prediction. Consistent with this idea, we note that inhibition of MKK7 mRNA splicing results in an elevated TNF-α response in T cells [57].

Increased inflammation in itself may not cause damage to the CNS, so long as the levels of anti- and pro-inflammatory cytokines and chemokines are regulated in a co-ordinated manner. Unbalanced upregulation of either anti- or pro-inflammatory cytokines may disrupt the intricate balance usually maintained throughout normal neurodevelopment. Of the immune molecules differentially regulated in *Map2k7* Hz mice compared to WTs, one is anti-inflammatory (IL-10) and three are pro-inflammatory (TNF-α, IL-2 and IL-12). IL-6 is considered both anti- and pro-inflammatory. Therefore, it is conceivable for there to be an imbalance of pro- vs. anti-inflammatory cytokines in *Map2k7* Hz mice that have been exposed to viral infection. Overall, these results suggest that gestating *Map2k7* Hz mice have a less well-controlled/regulated immune response to viral infection.

The elevation in VEGF levels is of interest. Patients with schizophrenia have been reported to have higher plasma VEGF levels than controls [58] and lower brain (prefrontal cortex) levels [59]. Suppressed JNK signalling has generally been associated with negative effects on VEGF expression [60], so the elevated plasma levels in *Map2k7* Hz mice may be secondary to increased cytokine levels (for example IL-6 [61]).

### Embryonic brain

In embryonic brain, Meyer et al. [20] reported elevated levels of IL-1β, IL-6 and IL-10 at 3 or 6 h, which were to some extent dependent on the gestational day of polyI:C administration. Increased brain levels of IL-1β and IL-5, but not IL-6 or IL-10, were also reported 6 h after polyI:C at E9 [22]. Another investigation found IL-1β, CCL2, CXCL9, CXCL10 and VEGF to be increased in embryonic brain following maternal exposure to polyI:C at E16 [17]. Of the cytokines that were in a detectable range in the embryonic brain, we found an overall increase only in CXCL10 and CCL5 but not in VEGF in response to polyI:C exposure. Other studies that used 20 mg/kg polyI:C could either not detect CCL5 or it was not contained within the set of cytokines that they measured. Interestingly, in the current study, CCL2 was increased following polyI:C in maternal plasma and was detectable in embryonic brain but did not show an overall increase following maternal polyI:C, in contrast to Arrode-Bruses and Bruses (2012), who found CCL2 to be increased in foetal brain 6 h following PolyI:C exposure.

Maternal haploinsufficiency for *Map2k7* resulted in elevated basal CXCL10 levels in embryonic brain. This is consistent with evidence that JNK suppresses polyI:C-stimulated activation of CXCL10 in macrophages [62]. Embryonic brain from *Map2k7* Hz mothers also showed an increased CCL5 induction in response to polyI:C. This is in clear contrast to the lower CCL5 induction in plasma from *Map2k7* Hz mothers and emphasises the complexity of genetic effects on the innate immune response.

It could be proposed that paternal genotype, as well as maternal genotype, might influence the embryonic brain response to MIA. There is a literature on increased risk of schizophrenia with advancing paternal age, which has been interpreted to suggest an inheritance of paternal genetic or epigenetic risk factors, via mechanisms such as paternal imprinting [63, 64]. However, it has now become clear that, for schizophrenia, this is not in fact a paternally derived genetic or epigenetic risk, but rather an epiphenomenon, possibly linked to corresponding increases in maternal age [65]. Therefore, we consider it is safe to assume that it is the maternal genotype which is the pre-eminent factor at work.

Embryonic haploinsufficiency for *Map2k7* resulted in increased brain CCL5 production, and an elevated CCL2 and CXCL10 response, to polyI:C. While there are few previous studies of the role of JNK on chemokine responses of immune cells, to place these results in context, those available would have predicted a general reduction in CCL5 production due to haploinsufficiency for *Map2k7.* JNK inhibition (albeit using potentially non-selective pharmacological tools) reportedly dampens chemokine (CCL2/CCL5) production as a result of polyI:C exposure in T cells [66], and in microglial cells, JNK inhibition reduces polyI:C-stimulated induction of CCL5, but not CXCL10 [67]. The data reported here strongly implicate JNK signalling in the embryonic brain chemokine response to maternal infection and reveal a complex interplay between maternal and embryonic genotypes.

Altered peripheral CCL5 levels have been detected in patients with schizophrenia [68, 69], which is of interest considering the data presented here, and the genetic association between schizophrenia and JNK pathway genes. The role of CCL5 in the CNS is not well-characterised. It is thought to be produced by neurons, oligodendrocytes, astrocytes and microglia [70], and, apart from a role in neuroinflammation, evidence that CCL5 can modulate synaptic glutamate release [71] may be particularly relevant to schizophrenia risk. Patients with schizophrenia, and those at high risk of developing the disease, show abnormal cortical glutamate levels [72]. While various mechanisms may be involved, the dysregulation of brain chemokines is likely to produce long-term effects on CNS development.

### Placenta

The placental environment will be important in foetal chemokine production as a result of MIA. This is reinforced by the increased schizophrenia risk associated with preeclampsia [38]. It has been noted experimentally that placenta-specific deletion of IL-6 prevents MIA-induced elevation in CXCL10 of foetal brain and reduces foetal brain VEGF and bFGF levels [73], emphasising the importance of the placenta for communicating the effects of maternal infection to the foetus.

CXCL10 expression was enhanced in the placenta from *Map2k7* Hz mice relative to controls. This is similar to the observations in embryonic brain and reinforces the evidence that MKK7-JNK signalling negatively regulates the expression of this chemokine. CXCL10 is a key mediator in preeclampsia [40, 74] and in the innate immune response to *Toxoplasma Gondii* [39], two of the best established immune risk factors for schizophrenia [6, 37, 38], so this has implications for understanding gene × environment risk interactions in the disease. CXCL10 has a suppressive effect on developing GABAergic interneurons, reducing GAD65/67 expression [44], so increased CXCL10 levels are likely have a detrimental effect on this population of cells. Note that CXCL10 can also affect synaptic glutamate responses [44], so CXCL10 dysregulation may potentially exacerbate any latent dysfunction of glutamatergic transmission in subjects with genetic risk for schizophrenia. CXCL12 is another chemokine strongly linked to preeclampsia and the placental trophoblast response to infection [41, 75, 76]. In contrast to the situation with CXCL10, the placenta CXCL12 response to polyI:C was suppressed in *Map2k7* Hz mice. It is known that decreased CXCL12 impairs migration and developmental function of cortical GABAergic interneurons [45, 46], so a reduction in CXCL12 is likely to compound the negative effects of increased CXCL10 on these cells.

### Maternal effects

In GWAS studies of schizophrenia, the genetic variants detected, comparing patients with control subjects, explain only a small proportion of the genetic risk associated with the disease—the remainder is the so-called *missing heritability*. Any influence, on offspring disease risk, of maternal genetic risk factors, acting for example via the inter-uterine environment, manifests in case-control association studies as an offspring genotype effect at reduced penetrance [77–79]. There is increasing awareness that such maternal effects should be considered when interpreting GWAS data. In phenotypes obviously dependent on both offspring and maternal factors, where maternal and offspring single-nucleotide polymorphisms (SNPs) are compared for their influence on offspring phenotype, the maternal SNPs can show the greater effect size (e.g. [80]). Hence, there is the possibility that some of the missing heritability in psychiatric disease derives from the effect of maternal genetic risk factors influencing the uterine environment, during prenatal exposure to environmental risk factors. Recent evidence supports this proposal in the case of autism spectrum disorders [81], where of course there is also a strong link to MIA. Indeed, there is some evidence suggesting that risk of schizophrenia in offspring may be increased to a greater extent by maternal genotype as compared to paternal genotype [82]. Our data are consistent with this, currently under-considered, hypothesis. They show that an interaction between environmental factors and maternal genotype influences the exposure of the developing embryo to chemokines known to influence GABA interneurone development. Future studies can test whether maternal genotype operates in a similar fashion in clinical populations.

## Conclusions

Our results clearly reveal that MKK7-JNK signalling plays a role in the viral immune response and that disruption of MKK7-JNK signalling affects both maternal and foetal induction of cytokines and chemokines. This disruption can be detected at various levels of the maternal and foetal response—in the maternal plasma, in the placenta and in embryonic brain. While illuminating the complexity of the interaction of genetic factors with the immune response in MIA, our results may be especially important in revealing the currently under-emphasised influence of maternal genotype on the foetal response to MIA.

## Additional file

Additional file 1: **Figure S1.** Correlation between CXCL10 in placenta and embryo brain. Supplementary methods—Luminex assay details. **Table S1.** F values for ANOVAs (DOCX 168 kb)

## Abbreviations

CSFs: Colony-stimulating factors
ELISA: Enzyme-linked immunosorbent assay
GAD-67: Glutamic acid decarboxylase-67
Hz: Hemizygous
polyI:C: Polyriboinosinic-polyribocytidylic acid
SNPs: Single-nucleotide polymorphisms
WT: Wild-type
