# Supplementary material for: JNK signalling mediates aspects of maternal immune activation: importance of maternal genotype in relation to schizophrenia risk
Source: J Neuroinflammation. 2019 Jan 28;16:18. doi: 10.1186/s12974-019-1408-5 (PMC6350402; doi:10.1186/s12974-019-1408-5)
Supplement: Supplementary file 1 — Figure S1. Correlation between CXCL10 in placenta and embryo brain. Supplementary methods—Luminex assay details. Table S1. F values for ANOVAs (DOCX 168 kb) [file 12974_2019_1408_MOESM1_ESM.docx]

**Figure S1**

Relationship between placental and embryonic brain levels of Cxcl10.


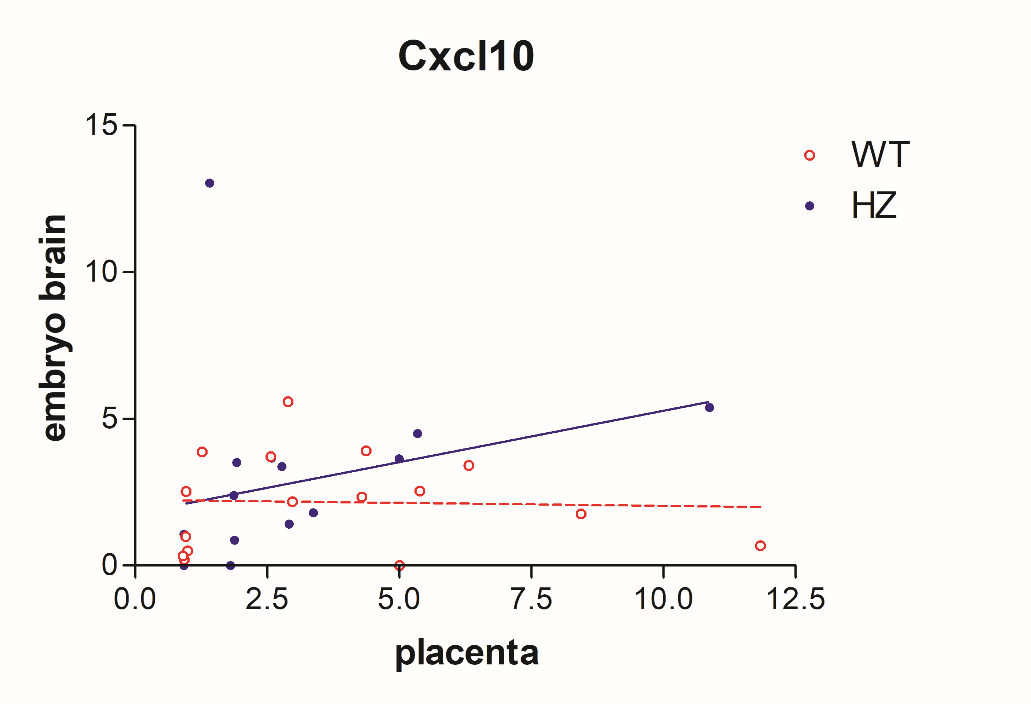


Correlation between placental and embryonic brain levels of Cxcl10, separated by embryo genotype

r^2^ = 0.01 (WT embryos) and 0.07 (Map2k7 Hz embryos).


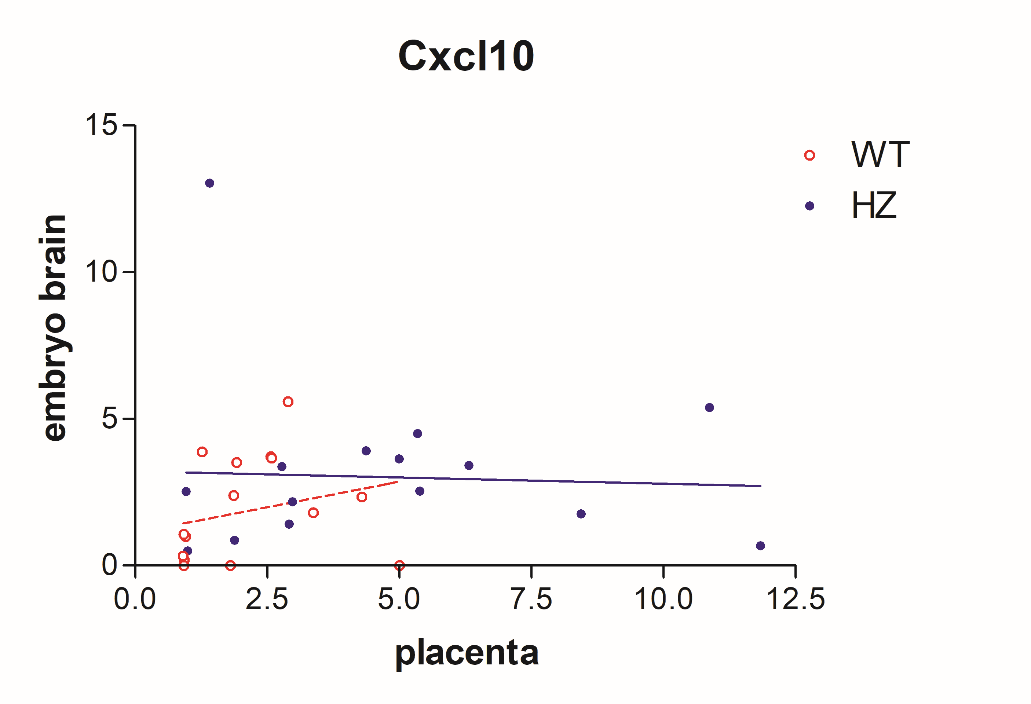


Correlation between placental and embryonic brain levels of Cxcl10, separated by maternal genotype

r^2^ = 0.07 (WT dams) and 0.01 (Map2k7 Hz dams).

**Supplementary Methods**

Luminex Assay

The protein molecules measured and the sensitivity of the assay is shown in Table S1. These three groups of immune molecules are involved in the first steps following viral infection and initiate the movement, transcription and release of various immune cells, therefore giving a broad overview of the maternal and embryonic reaction to infection.

**Cytokines Chemokines Growth factors**

TNF-α 23 IL-10 40 CXCL10 20 VEGF 6

IFN-γ 15 IL-12 8 CXCL1 68 FGFbasic (FGF2) 36

IL-1α 24 IL-13 24 CCL2 22

IL-1β 18 IL-17 7 CXCL9 7

IL-2 7 IL-5 22 CCL3 38

IL-4 40 IL-6 28

CSF2 16

*Table S1. Cytokines, chemokines and growth factors measured using the Luminex assay, with the lower limit of detection (in pg/ml). CSF2 = GM-CSF*

Antibody beads antibodies for each of the proteins in Table S1 were vortexed for 30 seconds then sonicated for 30 seconds immediately before they were added into each well (25μl of antibody bead solution per well). 50μl of incubation buffer was added into each well followed by the diluted standards in duplicate (100μl each). Samples were diluted 1:1 with assay diluent and 100μl of this was added per well, along with 50μl incubation buffer. The plate was then protected from light and incubated on an orbital shaker at 600 rpm overnight at 4°C. After further processing according to standard procedures, the plates were read on a recently calibrated and validated Bio-Plex® 200 MAGPIX multiplex reader (Bio-Rad, CA) using Bio-Plex Manager™ 5.0 software (Bio-Rad, CA). The appropriate bead region was assigned to each analyte, a detection target of 100 beads per region and the recommended doublet discriminator (DD) gates of 7,800 – 20,000 were used, and the median fluorescent intensity (MFI) was collected. The coefficient of variation (% CV) of duplicate wells was checked; a plate was acceptable if the mean CV<15%, and if not more than 20% of duplicates have CV>25%. The mean CV for each plate was 5.25% and 6.74% and the percentage of duplicates which have a CV >25% was 1.35% and 3.12% for each plate, respectively, which was well within this range. Any points from the standard curve that had a % CV >25% and accuracy outside 70-120% of expected values were excluded. % accuracy was calculated by the formula: (observed value/expected value)*100. The analysis software was then used to fit a curve to this set of reliable standards data using 5-parameter logistic regression with default automated weighting (all fitted to ≥ 6 points). Lower (Table 2) and upper limits of quantification (LLOQ and ULOQ) were calculated as the highest and lowest measured reliable standards for each standard curve after assessment as above. Concentration values that fall outside of this curve range were not included in analysis, unless they were out of range because of a group difference. For example, mice that had received polyI:C were likely to have cytokine levels within range but mice that had saline were not. In this case, the cytokine levels below detection threshold were given a value of 0.

| **Cytokine/Chemokine** | **WT vs. HZ**  **(Effect of Genotype)** | **Poly I:C vs. Saline**  **(Effect of Treatment)** | **WT with Poly I:C vs. HZ with Poly I:C (Genotype x Treatment interaction)** |
| --- | --- | --- | --- |
| CCL5 | 0.391, ns | <0.0001, F_(1,12)_=2249.70 | 0.397, ns |
| GM-CSF | NA | NA | NA |
| IFN-γ | NA | NA | NA |
| IL-1α | NA | NA | NA |
| IL-1β | 0.180, ns | 0.006, F_(1,12)_=11.11 | 0.344, ns |
| IL-2 | 0.008, F_(1,12)_=9.88 | <0.0001, F_(1,12)_=38.57 | 0.061, ns * |
| IL-4 | NA | NA | NA |
| IL-5 | 0.575, ns | <0.0001, F_(1,12)_=52.64 | 0.307, ns |
| IL-6 | 0.125, ns | <0.0001, F_(1,12)_=50.39 | 0.861, ns |
| IL-10 | 0.009, F_(1,12)_=9.78 | 0.002, F_(1,12)_=16.36 | 0.041, F_(1,12)_=5.23 |
| IL-12 | 0.057, F_(1,12)_=4.44 | <0.0001, F_(1,12)_=67.74 | 0.027, F_(1,12)_=6.35 |
| IL-13 | NA | NA | NA |
| IL-17 | NA | NA | NA |
| TNF-α | 0.026, F_(1,12)_=6.46 | <0.0001, F_(1,12)_=139.97 | 0.040, F_(1,12)_=5.33 |
| CXCL10 | 0.536, ns | <0.0001, F_(1,12)_=55.82 | 0.542, ns |
| CXCL1 | 0.050, F_(1,12)_=4.75 | <0.0001, F_(1,12)_=110.88 | 0.123, ns |
| CCL2 | 0.622, ns | <0.0001, F_(1,12)_=22.47 | 0.617, ns |
| CXCL9 | 0.289, ns | 0.004, F_(1,12)_=12.80 | 0.325, ns |
| CCL3 | NA | NA | NA |
| VEGF | 0.029, F_(1,12)_=6.19 | 0.251, ns | 0.157, ns |
| FGF basic | 0.311, ns | 0.433, ns | 0.255, ns |

**Table S2. p-values and F values from statistical tests showing significance between experimental groups of immune molecule levels in maternal plasma.**

| **Cytokine/Chemokine** | **Embryonic WT vs. HZ** | **Maternal WT vs. HZ** | **Poly I:C vs. Saline** | ***(Embryonic Genotype x Drug x Maternal Genotype interaction)** |
| --- | --- | --- | --- | --- |
| CCL5 | 0.771, ns | 0.031, F_(1,31)_=5.95 | <0.0001, F_(1,31)_=46.20 | 0.063, ~ F_(1,31)_= 4.20 |
| CXCL10 | 0.366, ns | 0.084, ns | <0.0001, F_(1,31)_=22.58 | 0.105, ns |
| CCL2 | 0.292, ns | 0.944, ns | 0.738, ns | 0.262, ns |
| VEGF | 0.278, ns | 0.565, ns | 0.651, ns | 0.616, ns |
| FGF basic | 0.123, ns | 0.213, ns | 0.178, ns | 0.183, ns |

**Table S3. p-values and F values from statistical tests showing significance between experimental groups of immune molecule levels in embryonic brain.**

| **Chemokine** | **Embryonic WT vs. HZ** | **Maternal WT vs. HZ** | **Poly I:C vs. Saline** | **(Drug x Maternal Genotype interaction)** |
| --- | --- | --- | --- | --- |
| CXCL10 | 0.332 ns | O.009, F_(1,34)_ = 9.06 | O.043, F_(1,34)_ = 4.88 | 0.375 ns |
| CXCL12 | 0.803 ns | 0.257, ns | 0.010, F_(1,31)_ = 8.66 | 0.017 F_(1,31)_ = 7.13 |

**Table S4. p-values and F values from statistical tests showing significance between experimental groups of immune molecule levels in placenta.**
